# Alteration of the α-Synuclein Folding Landscape by a Mutation Related to Parkinson’s Disease**

**DOI:** 10.1002/anie.201000378

**Published:** 2010-04-06

**Authors:** Allan Chris M Ferreon, Crystal R Moran, Josephine C Ferreon, Ashok A Deniz

**Affiliations:** * Department of Molecular Biology, The Scripps Research Institute10550 North Torrey Pines MB-19, La Jolla, CA 92037 (USA), Fax: (+1)858-784-9067 http://www.scripps.edu/mb/deniz

**Keywords:** conformational switching, FRET, protein folding, single-molecule biophysics, thermodynamics

α-Synuclein is an abundant presynaptic protein that is associated with several biological activities, including vesicle trafficking,^1^ maintenance of synaptic SNARE complexes and vesicle pools,^2^,^3^ and regulation of dopamine metabolism.^4^ The protein is believed to play a central role in the pathogenesis of Parkinson’s disease (PD), as evidenced by the identification of single point mutations and gene duplication/triplication that are causally linked to early-onset familial PD,^5^–^8^ as well as its conspicuous aggregation in filamentous inclusions associated with PD and other α-synucleinopathies.^9^ Under physiological conditions in vitro, α-synuclein exhibits properties of an intrinsically disordered protein (IDP).^10^ Like many other IDPs,^11^ α-synuclein is known to acquire structure upon binding to biological partners, with strong implications for the regulation of its function and dysfunction.^10^,^12^

Biological and biophysical studies have provided strong evidence to suggest an important link between the lipid/membrane binding of α-synuclein and its physiological role.^13^–^16^ Several NMR, EPR, CD, fluorescence, and other biophysical studies have demonstrated that binding to membranes and lipid mimics confers helicity on the protein,^10^,^14^–^21^ and that different conditions or binding partners can promote the adoption of bent or extended helical structures,^10^,^14^–^22^ although different studies have sometimes led to contrasting views as to which of the two structures is populated by the protein on biological membranes.

Recently, single-molecule methods have been applied in structural studies of α-synuclein.^16^,^22^–^25^ Single-molecule measurements avoid the signal averaging inherent in conventional ensemble studies and can therefore reveal hidden information in complex systems such as α-synuclein and other IDPs,^26^,^27^ which are in general prone to structural heterogeneity. Our recent single-molecule FRET (smFRET; FRET=Förster resonance energy transfer) study shed new light on the folding characteristics of wild-type (WT; 140 residues) α-synuclein as induced by binding to small unilamellar vesicles (SUVs), and membrane and lipid mimetics.^16^ That study in combination with previous ensemble thermodynamics measurements clearly established that the bent and helical α-synuclein structures could coexist in the presence of the same binding partners.^10^ More surprisingly, similar dual-structural ensembles were found to be populated not only in the presence of membranelike surfaces, but also in association with small-molecule ligands. The aggregation characteristics of α-synuclein appear to be dependent on the conformational state of the monomeric protein;^10^ thus, the complex interplay between binding and folding is directly relevant to the disease aspects of the protein.

In this study, we probed the effects of a few key mutations on the coupled binding and folding of α-synuclein by using a combination of single-molecule (smFRET) and ensemble (far-UV CD) measurements. Specifically, we examined the PD-linked single-residue mutations A30P, A53T, and E46K.^5^–^7^ We also tested a deletion mutant, αSyn 1–107, to probe the role of the C-terminal tail in the binding–folding behavior of α-synuclein. Our results show that the A53T, E46K, and C-terminal-truncation mutations do not lead to loss of the multistate folding character of the WT protein.^10^ In contrast, the A30P mutation energetically favors population of the bent helix conformation.

Under the solution conditions used, all the α-synuclein mutants studied exhibited far-UV CD spectra consistent with structures that are predominantly random coil (see Figure S1 in the Supporting Information);^28^ that is, they are intrinsically unstructured under dilute buffer conditions and in the absence of ligands, similarly to the WT.^10^ We then employed sodium dodecyl sulphate (SDS) as a lipid mimetic and small-molecule amphiphilic ligand, and used it to induce ordered structures in the WT and mutant proteins. We monitored secondary structural changes by CD spectroscopy. Under the experimental conditions used, the SDS critical micelle concentration (CMC) is approximately 1 mm.^10^ At 10 mm SDS, a concentration at which SDS molecules exist predominantly as spherical micelles, WT, A30P, and E46K α-synuclein had practically identical helicities of about 63%, whereby the percentage α-helix values were estimated on the basis of protein far-UV CD spectra, as previously described (see Figure S2A,B,D in the Supporting Information).^28^ By comparison, A53T and αSyn 1–107 were somewhat more helical (ca. 69 and 76%, respectively; see Figure S2C,E in the Supporting Information). A decrease in the SDS concentration to 1 mm so that SDS was mostly monomeric resulted in increased helicity in all variants (WT, ca. 77%; A53T, ca. 87%; E46 K, ca. 80%; αSyn 1–107, ca. 93%) except for A30P (ca. 54%).

**Figure 1:**
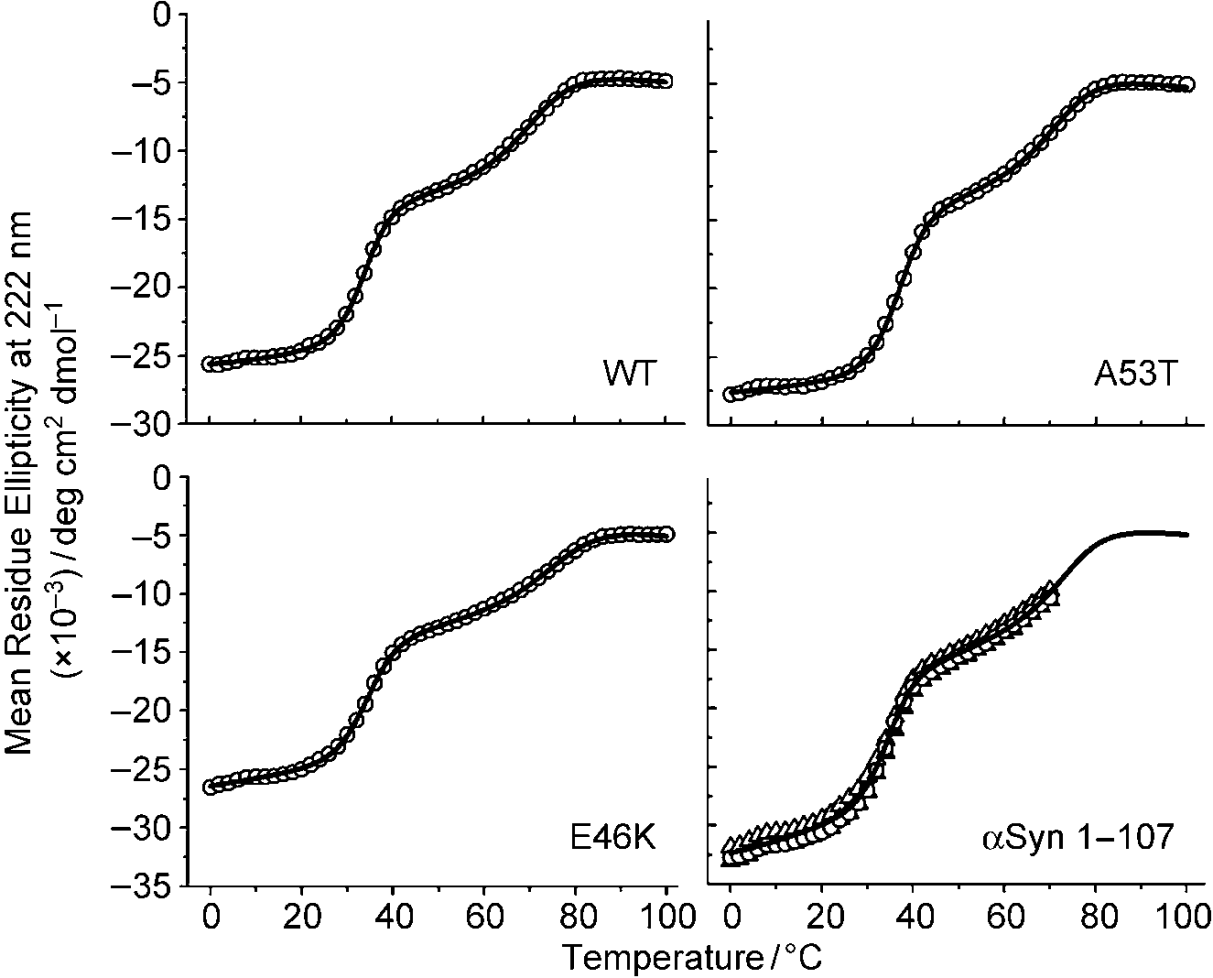
Three-state thermal unfolding of WT α-synuclein, the PD-linked A53T and E46K mutants, and a C-terminal-truncation variant (residues 1–107). Protein-denaturation transitions were monitored by far-UV CD spectroscopy in the presence of SDS (1 mm). Unfolding data were collected from 0 to 100°C for all peptides except αSyn 1–107 (0–70°C), which aggregates at higher temperatures under the experimental conditions used (see the Supporting Information for additional details).

**Figure 2:**
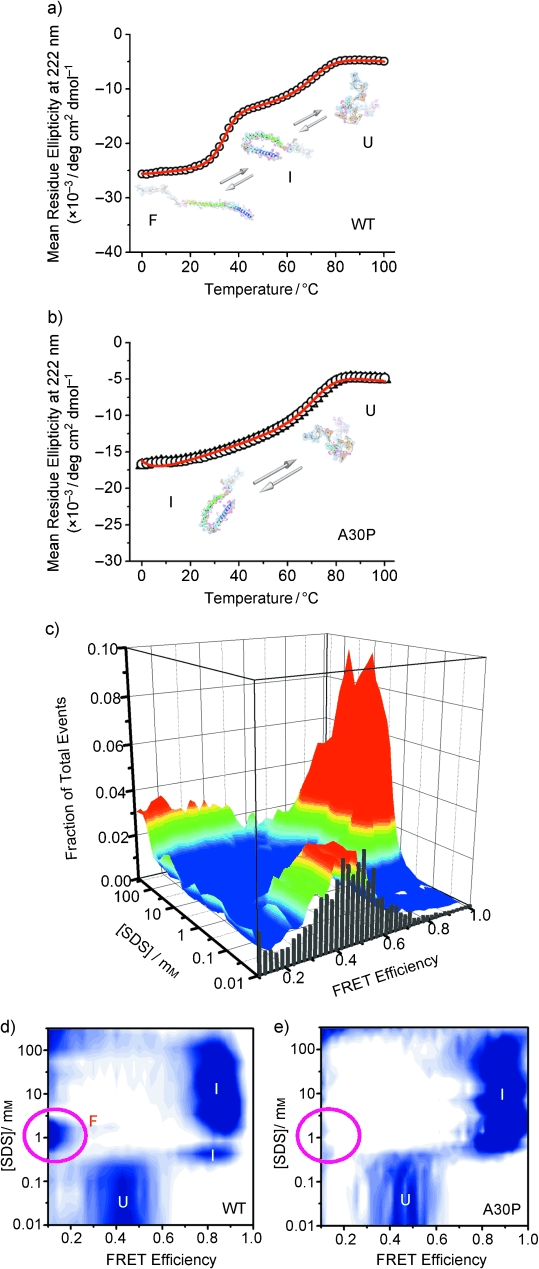
Single-molecule and ensemble characterization of the effect of the PD-linked A30P mutation on the coupled binding and folding of α-synuclein. a,b) Thermal unfolding of WT α-synuclein (a) and the A30P mutant (b) bound to SDS monomers, as monitored at the ensemble level by CD spectroscopy in the presence of SDS (1 mm). c) Conformational transitions of A30P α-synuclein induced by changes in ligand concentration at room temperature, as detected at the single-molecule level by smFRET. d) Two-dimensional contour plot of previously reported WT data^16^ for comparison with the corresponding plot for the A30P mutant in (e). e) Two-dimensional contour plot of the raw data presented in (c). U, I, and F are different protein conformational states that exhibit characteristic FRET efficiency^16^ and helicity.^10^ U is the natively unfolded state, I corresponds to a bent helical structure previously solved by NMR spectroscopy (1XQ8),^18^ and F is an extended α-helix structure. (See the Supporting Information for additional details).

To further study the effects of mutation on the binding-induced folding characteristics of α-synuclein, we identified temperature-induced conformational transitions for the different variants by monitoring ellipticity at 222 nm (Figure 1; see also Figure S3A–E in the Supporting Information). The thermal-denaturation data were analyzed by using the model described previously^10^ (the derived unfolding-transition thermodynamic parameters are summarized in Table S1 in the Supporting Information). The three experimentally observed conformational states were denoted as U, F, and I: U is the intrinsically disordered “unfolded” state, F is the “folded” state that is most α-helical, and I is the ordered state exhibiting “intermediate” helicity. At an SDS concentration of 1 mm, the A53T and E46K mutants maintained the three-state thermal-unfolding behavior exhibited by WT α-synuclein (Figures 1 and 2a) and populated the F conformational state in the temperature range investigated. Furthermore, C-terminal truncation of the protein resulted in a minimal effect on its coupled folding–binding properties (Figure 1), which suggests that the highly basic N-terminal and hydrophobic NAC (non-Aβ component of Alzheimer’s disease amyloid) regions of α-synuclein can interact with ligands/membranes independently of the highly acidic C-terminal tail region. In sharp contrast, A30P α-synuclein showed strikingly different behavior consistent with a two-state transition (Figure 2b).

To further investigate this altered A30P folding, we carried out smFRET experiments with a home-built microscope system described previously.^16^ For the smFRET measurements, donor (D; Alexa Fluor 488) and acceptor (A; Alexa Fluor 594) FRET probes were attached to A30P α-synuclein through the reaction of maleimide dyes with cysteine residues incorporated by site-directed mutagenesis at positions 7 and 84 (see the Supporting Information). The dyes were placed at identical locations in the A30P and WT proteins, and the A30P smFRET data (Figure 2c,e) are entirely consistent with data from the CD measurements on unlabeled protein (Figure 2b), as previously noted for the WT protein.^10^,^16^ Hence, the dye labeling does not significantly perturb the folding–binding properties of the A30P mutant. During smFRET experiments performed over a wide range of SDS concentrations (0–400 mm), D and A bursts were detected simultaneously while individual labeled protein molecules diffused freely through a diffraction-limited sub-fL confocal detection volume. These signals were used to calculate single-molecule FRET efficiencies (*E*_FRET_) in the same manner as previously described.^16^

In Figure 2c is a histogram depicting the fraction of events plotted against measured *E*_FRET_ values and ligand concentration. The same data are also plotted as a 2D contour plot in Figure 2e. For comparison, the published WT smFRET data were replotted in an identical manner (Figure 2d).^16^ A clear difference between the WT and A30P data is the absence of the F-state peak at low *E*_FRET_ values in the A30P data at SDS concentrations that favor the SDS monomer (i.e., near and below the CMC of ca. 1 mm). This result is consistent with the thermal-denaturation data presented in Figure 2a,b and suggests that for the A30P mutant, the bent helical structure of the I state is energetically favored over the extended helical structure of the F state. At very high SDS concentrations (>100 mm), at which SDS forms cylindrical micelles, population density at low *E*_FRET_ values indicates that A30P α-synuclein may be capable of adopting more extended structures under some conditions, although they are not preferred conformations.

The data presented herein demonstrate that in contrast with the other mutations studied, the A30P mutation significantly perturbs the binding–folding thermodynamics of α-synuclein and causes the protein to inherently favor the “I-state” bent helical structure over the “F-state” extended helix structure. The conformational variance observed between the protein mutants indicates that different molecular disease mechanisms may be responsible for the different early-onset variants of PD, a tentative possibility awaiting more-direct future testing. Our data clearly show that the A30P α-synuclein conformational properties are significantly altered, probably as a result of the strong helix-breaking propensity of the proline substitution. Future higher-resolution studies could reveal more-subtle conformational differences for the other mutants as well. Our A30P results, in conjunction with previous studies that demonstrated a reduced affinity of the A30P mutant for membranes in vivo^13^,^29^ and in vitro,^30^ provide additional support for the hypothesis that the F-state extended helical structure is the primary membrane-binding-competent α-synuclein species.^16^,^17^,^21^,^22^,^31^ The observed conformational propensities of the A30P mutant may also contribute to its lowered partitioning into lipid rafts.^32^ Furthermore, the reduced ability of A30P α-synuclein to form the F conformation, when taken together with the observation that the A30P mutation decreases the rate of fibrillation (and increases the rate of nonfibrillar oligomer formation),^33^ represents new evidence in support of the importance of the F state as a fibrillation intermediate.^10^ Finally, our observations of altered A30P behavior in the absence of membrane-like surfaces are significant because the majority of α-synuclein expressed in vivo exists as soluble protein not bound to membranes;^34^ therefore, the regulation of protein structure and function in the soluble monomeric form is an important object of study.

To conclude, this structural-thermodynamics study presents a striking demonstration of a clear, molecular consequence of a PD-related mutation. Such changes to IDP binding–folding landscapes may be linked to disease either through loss of function or gain of toxicity. Our results are likely to contribute to future investigations aimed at further understanding the underlying biophysical chemistry and molecular mechanism(s) of PD and other α-synucleinopathies, as well as the native function of α-synuclein. Finally, our study also showcases the potential of single-molecule experiments to explore and reveal critical structural intricacies in complex folding landscapes for IDPs and amyloid-forming proteins in general.
