# Supplementary material for: Alteration of the α-Synuclein Folding Landscape by a Mutation Related to Parkinson’s Disease
Source: Angew Chem Int Ed Engl. 2010 Apr 6;49(20):3469–72. doi: 10.1002/anie.201000378 (PMC2972640; doi:10.1002/anie.201000378)
Supplement: Supplementary file 1 [file anie0049-3469-SD1.pdf]

Angewandte Chemie
Eine Zeitschrift der Gesellschaft Deutscher Chemiker

Supporting Information
© Wiley-VCH 2010
69451 Weinheim, Germany

Alteration of the α-Synuclein Folding Landscape by a Mutation Related to Parkinson's Disease**

*Allan Chris M. Ferreon, Crystal R. Moran, Josephine C. Ferreon, and Ashok A. Deniz**

anie_201000378_sm_miscellaneous_information.pdf

SUPPORTING INFORMATION

MATERIALS AND METHODS

Chemicals. Ultrapure sodium dodecyl sulfate (SDS) was purchased from USB Corp. (lot 115177). All other chemicals used were of either analytical or reagent grade.

Protein Expression and Purification. Variants of the 140-residue α-synuclein (G7C/G84C; A30P; G7C/G84C/A30P; A53T; E46K; and, C-terminal truncation [res. 1-107]) were prepared through QuickChange site-directed mutagenesis (Stratagene, Garden Grove, CA) starting from a wildtype α-synuclein plasmid construct that was kindly provided by Dr. Robert L. Nussbaum (National Institutes of Health). The expression and purification of wildtype and mutant α-synuclein proteins were performed on the basis of previously described protocols,[1, 2] employing high temperature or ammonium sulfate precipitation, and reverse phase HPLC and/or ion exchange chromatography. The purity of the final protein products was verified by SDS-PAGE. Protein molecular weights were verified using mass spectrometry (Scripps Center for Mass Spectrometry).

Protein Labeling for FRET Experiments. G7C/G84C/A30P α-synuclein labeling was carried out overnight at 4ºC in 6 M guanidine hydrochloride, 50 mM Tris-HCl, pH 7.2 using a 1:1.25:7 ratio of protein, Alexa Fluor 488 maleimide (donor; Molecular Probes, Carlsbad, CA) and Alexa Fluor 594 maleimide (acceptor; Molecular Probes), respectively. Three rounds of ethanol precipitation were performed to separate free dyes from the labeled protein. The molecular weight of the labeled product was verified by mass spectrometry.

Protein Folding/Unfolding Detected by Far-UV CD Spectroscopy. SDS- or temperature-induced structural changes in wildtype and mutant α-synuclein variants were monitored in 0.2 M NaCl, 10 mM sodium acetate, 10 mM NaH_2PO_4 and 10 mM glycine, at pH 7.50 ± 0.05 using an Aviv Model 62DS/202SF CD spectrometer equipped with a Peltier automated temperature control unit. CD signals at specific wavelengths were expressed as mean residue ellipticity $[\theta]_\lambda$ (deg cm^2 dmol^{-1}), given by the expression $\{[\theta]_\lambda \equiv \theta_\lambda /(10Cnl)\}$, where θ_λ is the observed ellipticity in millidegrees at wavelength λ, C is the molar protein concentration, n is the number of amino acid residues per protein molecule, and l is the pathlength in centimeters. Wavelength and temperature CD scans were performed using a bandwidth of 1 nm, a minimum averaging time of 5 sec, and a 0.1 cm-pathlength cuvette maintained at specific temperatures with 0.1 °C precision. Experiments were performed using 20 μM protein concentration.

Thermal scans were carried out using a scan rate of ~1.7 °C/min. All measurements were corrected for solvent contributions. Nonlinear least-squares (NLS) analyses of thermal unfolding data were carried out using Origin (OriginLab), employing two-state or three-state thermodynamic models (described previously[1]). Protein concentrations were spectrophotometrically determined using the Edelhoch method.[3-5]

Binding-Induced Protein Folding Detected by Single-Molecule Fluorescence Resonance Energy Transfer (smFRET). The SDS-induced folding of FRET-labeled G7C, G84C, A30P α-synuclein in 0.2 M NaCl, 10 mM sodium acetate, 10 mM NaH_2PO_4 and 10 mM glycine, at pH 7.50 ± 0.05, room temperature, in the presence of 20 μM A30P α-synuclein was monitored using single-molecule spectroscopy. smFRET measurements and data analyses were performed as previously described.[6]

SI REFERENCES

[1] A. C. Ferreon, A. A. Deniz, *Biochemistry* **2007**, *46*, 4499.
[2] R. Jakes, M. G. Spillantini, M. Goedert, *FEBS Lett.* **1994**, *345*, 27.
[3] S. C. Gill, P. H. von Hippel, *Anal. Biochem.* **1989**, *182*, 319.
[4] H. Edelhoch, *Biochemistry* **1967**, *6*, 1948.
[5] C. N. Pace, F. Vajdos, L. Fee, G. Grimsley, T. Gray, *Protein Sci.* **1995**, *4*, 2411.
[6] A. C. Ferreon, Y. Gambin, E. A. Lemke, A. A. Deniz, *Proc. Natl. Acad. Sci. U S A* **2009**, *106*, 5645.

MAIN TEXT REFERENCES

[1] A. A. Cooper, A. D. Gitler, A. Cashikar, C. M. Haynes, K. J. Hill, B. Bhullar, K. Liu, K. Xu, K. E. Strathearn, F. Liu, S. Cao, K. A. Caldwell, G. A. Caldwell, G. Marsischky, R. D. Kolodner, J. Labaer, J. C. Rochet, N. M. Bonini, S. Lindquist, *Science* **2006**, *313*, 324.

[2] S. Chandra, G. Gallardo, R. Fernandez-Chacon, O. M. Schluter, T. C. Sudhof, *Cell* **2005**, *123*, 383.

[3] D. E. Cabin, K. Shimazu, D. Murphy, N. B. Cole, W. Gottschalk, K. L. McIlwain, B. Orrison, A. Chen, C. E. Ellis, R. Paylor, B. Lu, R. L. Nussbaum, *J. Neurosci.* **2002**, *22*, 8797.

[4] A. Sidhu, C. Wersinger, P. Vernier, *FASEB J.* **2004**, *18*, 637.

[5] M. H. Polymeropoulos, C. Lavedan, E. Leroy, S. E. Ide, A. Dehejia, A. Dutra, B. Pike, H. Root, J. Rubenstein, R. Boyer, E. S. Stenroos, S. Chandrasekharappa, A. Athanassiadou, T. Papapetropoulos, W. G. Johnson, A. M. Lazzarini, R. C. Duvoisin, G. Di Iorio, L. I. Golbe, R. L. Nussbaum, *Science* **1997**, *276*, 2045.

[6] R. Kruger, W. Kuhn, T. Muller, D. Woitalla, M. Graeber, S. Kosel, H. Przuntek, J. T. Epplen, L. Schols, O. Riess, *Nat. Genet.* **1998**, *18*, 106.

[7] J. J. Zarranz, J. Alegre, J. C. Gomez-Esteban, E. Lezcano, R. Ros, I. Ampuero, L. Vidal, J. Hoenicka, O. Rodriguez, B. Atares, V. Llorens, E. Gomez Tortosa, T. del Ser, D. G. Munoz, J. G. de Yebenes, *Ann. Neurol.* **2004**, *55*, 164.

[8] A. B. Singleton, M. Farrer, J. Johnson, A. Singleton, S. Hague, J. Kachergus, M. Hulihan, T. Peuralinna, A. Dutra, R. Nussbaum, S. Lincoln, A. Crawley, M. Hanson, D. Maraganore, C. Adler, M. R. Cookson, M. Muenter, M. Baptista, D. Miller, J. Blancato, J. Hardy, K. Gwinn-Hardy, *Science* **2003**, *302*, 841.

[9] M. G. Spillantini, M. L. Schmidt, V. M. Lee, J. Q. Trojanowski, R. Jakes, M. Goedert, *Nature* **1997**, *388*, 839.

[10] A. C. Ferreon, A. A. Deniz, *Biochemistry* **2007**, *46*, 4499.

[11] P. E. Wright, H. J. Dyson, *Curr. Opin. Struct. Biol.* **2009**, *19*, 31.

[12] W. S. Woods, J. M. Boettcher, D. H. Zhou, K. D. Kloepper, K. L. Hartman, D. T. Ladror, Z. Qi, C. M. Rienstra, J. M. George, *J. Biol. Chem.* **2007**, *282*, 34555.

[13] P. H. Jensen, M. S. Nielsen, R. Jakes, C. G. Dotti, M. Goedert, *J. Biol. Chem.* **1998**, *273*, 26292.

[14] S. Chandra, X. Chen, J. Rizo, R. Jahn, T. C. Sudhof, *J. Biol. Chem.* **2003**, *278*, 15313.

[15] R. Bussell, Jr., D. Eliezer, *J. Mol. Biol.* **2003**, *329*, 763.

[16] A. C. Ferreon, Y. Gambin, E. A. Lemke, A. A. Deniz, *Proc. Natl. Acad. Sci. U S A* **2009**, *106*, 5645.

[17] C. C. Jao, A. Der-Sarkissian, J. Chen, R. Langen, *Proc. Natl. Acad. Sci. USA* **2004**, *101*, 8331.

[18] T. S. Ulmer, A. Bax, N. B. Cole, R. L. Nussbaum, *J. Biol. Chem.* **2005**, *280*, 9595.

[19] M. Drescher, G. Veldhuis, B. D. van Rooijen, S. Milikisyants, V. Subramaniam, M. Huber, *J. Am. Chem. Soc.* **2008**, *130*, 7796.

[20] M. Bortolus, F. Tombolato, I. Tessari, M. Bisaglia, S. Mammi, L. Bubacco, A. Ferrarini, A. L. Maniero, *J. Am. Chem. Soc.* **2008**, *130*, 6690.

[21] E. R. Georgieva, T. F. Ramlall, P. P. Borbat, J. H. Freed, D. Eliezer, *J. Am. Chem. Soc.* **2008**, *130*, 12856.

[22] A. J. Trexler, E. Rhoades, *Biochemistry* **2009**, *48*, 2304.

[23] G. Veldhuis, I. Segers-Nolten, E. Ferlemann, V. Subramaniam, *Chembiochem* **2009**, *10*, 436.

[24] M. Brucale, M. Sandal, S. Di Maio, A. Rampioni, I. Tessari, L. Tosatto, M. Bisaglia, L. Bubacco, B. Samori, *Chembiochem* **2009**, *10*, 176.

[25] J. Yu, S. Malkova, Y. L. Lyubchenko, *J. Mol. Biol.* **2008**, *384*, 992.

[26] F. Hillger, D. Hänni, D. Nettels, S. Geister, M. Grandin, M. Textor, B. Schuler, *Angewandte Chemie International Edition* **2008**, *47*, 6184.

[27] M. Bertz, A. Kunfermann, M. Rief, *Angewandte Chemie International Edition* **2008**, *47*, 8192.

[28] N. Greenfield, G. D. Fasman, *Biochemistry* **1969**, *8*, 4108.

[29] N. B. Cole, D. D. Murphy, T. Grider, S. Rueter, D. Brasaemle, R. L. Nussbaum, *J. Biol. Chem.* **2002**, *277*, 6344.

[30] R. Bussell, Jr., D. Eliezer, *Biochemistry* **2004**, *43*, 4810.

[31] C. C. Jao, B. G. Hegde, J. Chen, I. S. Haworth, R. Langen, *Proc. Natl. Acad. Sci. U S A* **2008**, *105*, 19666.

[32] D. L. Fortin, M. D. Troyer, K. Nakamura, S. Kubo, M. D. Anthony, R. H. Edwards, *J. Neurosci.* **2004**, *24*, 6715.

[33] K. A. Conway, S. J. Lee, J. C. Rochet, T. T. Ding, R. E. Williamson, P. T. Lansbury, Jr., *Proc. Natl. Acad. Sci. U S A* **2000**, *97*, 571.

[34] J. M. George, H. Jin, W. S. Woods, D. F. Clayton, *Neuron* **1995**, *15*, 361.

Table S1: Multistate thermal unfolding thermodynamic parameters in 1 mM SDS of wildtype α-synuclein and its mutants[a]

Protein	$T_m^{F\text{-}I}$ (°C)	$\Delta H^{F\text{-}I}$ (kcal/mol)	$\Delta Cp^{F\text{-}I}$ (kcal/mol-°C)	$T_m^{I\text{-}U}$ (°C)	$\Delta H^{I\text{-}U}$ (kcal/mol)	$\Delta Cp^{I\text{-}U}$ (kcal/mol-°C)
WT	33.3 ± 0.2	62.2 ± 2.6	2.0 ± 0.8	72.0 ± 0.2	47.8 ± 1.5	1.2 ± 0.3
A30P[b]	N.A.	N.A.	N.A.	71.7 ± 0.2	44.5 ± 2.0	1.0 ± 0.1
A53T	36.2 ± 0.2	57.2 ± 2.8	1.8 ± 0.6	72.3 ± 0.4	49.0 ± 2.8	1.1 ± 0.6
E46K	33.8 ± 0.3	55.3 ± 2.9	1.6 ± 0.7	76.4 ± 0.4	44.4 ± 2.2	1.0 ± 0.4
αSyn 1-107[c]	33.9 ± 0.3	59.1 ± 4.8	N.D.	74.2 ± 0.7	50.0 ± 6.8	N.D.

[a] Errors reported are estimated standard errors (Origin); N.A. - not applicable; N.D. - not determined
[b] NLS fitting performed simultaneously on two independent measurements, sharing all parameters
[c] NLS fitting performed simultaneously on four independent measurements, sharing all parameters; $\Delta Cp^{F\text{-}I}$ and $\Delta Cp^{I\text{-}U}$ parameters were fixed to the values determined for the wildtype protein

FIGURE S1

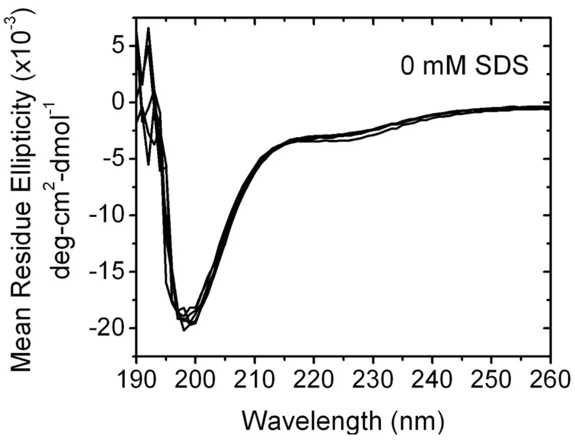

Wildtype and mutant α-synuclein proteins are predominantly unstructured in dilute buffer conditions. Far-UV CD spectra were recorded for the WT, A30P, A53T, E46K and αSyn 1-107 proteins at 25°C in the absence of the lipid mimetic SDS. *See SI Materials and Methods for additional details.*

FIGURE S2

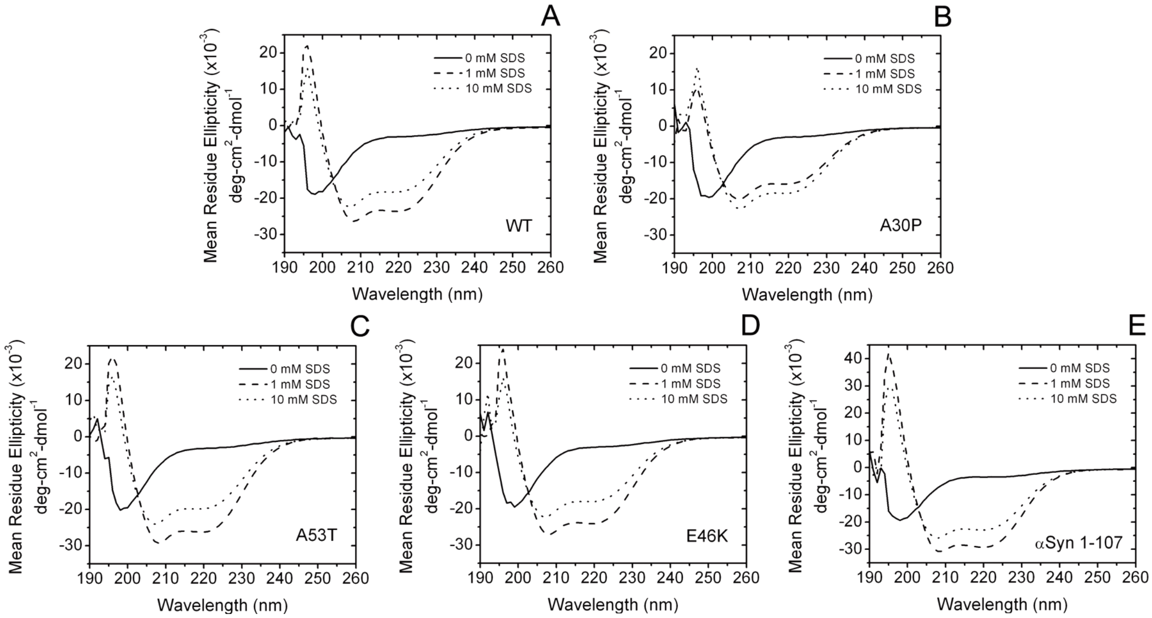

A30P mutation perturbs α-synuclein interaction with SDS monomers and micelles. Far-UV CD spectra were collected at 25°C in the presence of different concentrations of SDS. At the solution conditions used, SDS is primarily monomeric at concentrations ≤ 1 mM and micellar at 10 mM, and exhibits a critical micelle concentration of ~1 mM[1]. *See SI Materials and Methods for additional details.*

FIGURE S3

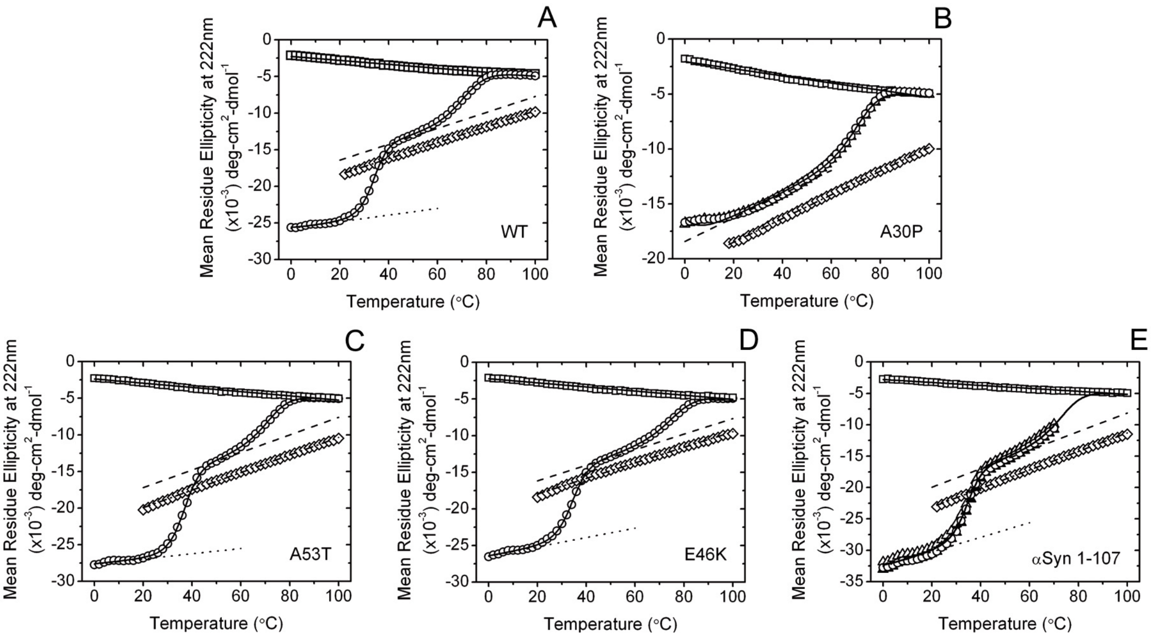

A53T, E46K and C-terminal truncation mutations maintain the three-state folding behavior of α-synuclein; A30P α-synuclein exhibits two-state character. Thermal denaturation measurements were performed in the presence of 0 (□), 1 (○,△) and 10 (◇) mM SDS, monitoring changes in protein secondary structure by detecting ellipticities at 222 nm. Straight lines represent linear fits to the 0 or 10 mM SDS data, or the NLS best fit baselines. The solid nonlinear curves are the NLS best fits of the 1 mM data to three-state (panels A and C-E) or two-state (panel B) thermal unfolding models[1]. *See SI Materials and Methods for additional details.*
